# A Qualitative Exploration of Student Cognition When Answering Text-Only or Image-Based Histology Multiple-Choice Questions

**DOI:** 10.1007/s40670-024-02104-x

**Published:** 2024-07-24

**Authors:** Jane Holland, Alice McGarvey, Michelle Flood, Pauline Joyce, Teresa Pawlikowska

**Affiliations:** 1 Department of Anatomy and Regenerative Medicine, RCSI University of Medicine and Health Sciences, Dublin, D02 YN77 Ireland; 2 Pharmacy and Biomolecular Sciences, RCSI University of Medicine and Health Sciences, Dublin, D02 YN77 Ireland; 3 Physician Associate Program, RCSI University of Medicine and Health Sciences, Dublin, D02 YN77 Ireland; 4 Health Professions Education Centre, RCSI University of Medicine and Health Sciences, Dublin, D02 YN77 Ireland

**Keywords:** Assessment, Histology, Images, Multiple-choice questions, Cognitive load

## Abstract

Interpretation of images and spatial relationships is essential in medicine, but the evidence base on how to assess these skills is sparse. Thirty medical students were randomized into two groups (A and B), and invited to “think aloud” while completing 14 histology MCQs. All students answered six identical MCQs, three with only text and three requiring image interpretation. Students then answered eight “matched” questions, where a text-only MCQ on version A was “matched” with an image-based MCQ on paper B, or vice versa. Students’ verbalizations were coded with a realist, inductive approach and emerging codes were identified and integrated within overarching themes. High-performing students were more likely to self-generate an answer as compared to middle and lower performing students, who verbalized more option elimination. Images had no consistent influence on item statistics, and students’ self-identified visual-verbal preference (“learning style”) had no consistent influence on their results for text or image-based questions. Students’ verbalizations regarding images depended on whether interpretation of the adjacent image was necessary to answer the question or not. Specific comments about the image were present in 95% of student-item verbalizations (142 of 150) if interpreting the image was essential to answering the question, whereas few students referred to images if they were an unnecessary addition to the vignette. In conclusion, while assessing image interpretation is necessary for authenticity and constructive alignment, MCQs should be constructed to only include information and images relevant to answering the question, and avoid adding unnecessary information or images that may increase extraneous cognitive load.

**Supplementary Information:**

The online version contains supplementary material available at 10.1007/s40670-024-02104-x.

## Introduction

Visuospatial skills are an intrinsic element of medicine and medical sciences, and the disciplines of anatomy and histology are typically the first areas of the medical curriculum where students will experience the need to develop and display skills of identification and interpretation. How these skills are introduced, internalized, and ultimately assessed has direct relevance to educators, particularly as technological advances have led to image-based resources and assessments being more easily, and so increasingly, incorporated into curricula.

Much education has moved online in recent years, with many institutions now teaching histology by means of virtual microscopy or computer-based programs [1–7]. The use of images in teaching and learning is well described, where the *dual-channels assumption* of the *multiple representation principle* proposes that learners process information primarily through separate auditory-verbal and visual-pictorial channels [8–11]. Students will have differences in cognitive ability, learning styles, and preferences along the visualizer–verbalizer dimension, and the concept of teaching to learning styles or preferences is still pervasive in education [12–14]. However, there is a dearth of evidence to support teaching to individual learning styles or preferences [15, 16], with “no adequate evidence base to justify incorporating learning styles assessments into general educational practice” [17]. Instead, the evidence base demonstrates that students benefit from learning with a combination of images and verbal information, to balance incoming information between these two main channels [18, 19].

Information on the effect of images in assessments is more limited, perhaps in part due to the historical challenges in preparing and including images in unique examinations. Nowadays, digital photography, printing, and online assessments mean that image reproduction and inclusion has become a straightforward task [20–24]. While all assessment methods have different strengths and weaknesses, multiple-choice questions (MCQs) are extremely time-efficient, allowing broad sampling across the curriculum, and so remain a core component of most programs of assessment [25–27]. Medicine and the medical sciences require accurate identification of clinical signs, anatomical parts, and histological features, and while precise verbal descriptions could be included within clinical vignettes, doing so may make text overly grammatically convoluted or complex [25, 27–29]. This issue is even more relevant for institutions with substantial numbers of non-native English speakers, studying medicine in their second (or third) language, and so conscious consideration should be given to only include *construct-relevant* language, an inherent part of the technical vocabulary of medical sciences, while minimizing linguistic clutter and irrelevant grammatical complexity [10, 27, 30].

Many learning outcomes in histology also require that students identify structures by visual inspection and interpretation [31]. While principles of constructive alignment require that these learning outcomes be assessed using images, the evidence base on how to do so is sparse, with variable outcomes [23, 32]. The bespoke 70-plate booklet of illustrations used by Hunt et al. in the 1970s was undoubtedly of high quality, and improved the authenticity of the assessment, but its use negatively impacted candidates’ scores, as they repeatedly switched focus between this booklet and reading the questions on the examination paper [20]. This phenomenon is described as the *spatial continuity* effect and can be avoided by placing text and images adjacent to each other in either printed or online assessments [9, 33]. More recent studies with well-aligned text and images found no evidence of a *consistent* effect on item difficulty or discrimination [24, 34, 35].

So, where lies the balance within authentic assessment of an undergraduate histology curriculum? Inclusion of some images within assessments is now a simple matter, but does an accompanying image provide candidates with an additional advantage or cue when answering a question, or is it a distracting increase to cognitive load [36, 37]? This study aims to address this gap by investigating how images influence medical students’ reasoning in histology MCQs, specifically (1) the cognitive processes and critical thinking of students while answering single best answer MCQs in histology, (2) whether images influence the verbalized cognitive processes of participants, and (3) whether self-identified verbal and visual learners display different verbalizations or cognitive processes when answering text and image-based MCQs.

## Materials and Methods

### Ethics Approval, Student Recruitment, and Anonymization

Ethical approval for this study was received from the Research Ethics Committee of the Royal College of Surgeons in Ireland (reference RCSI-REC1132). Students in their first year of both the Direct (Undergraduate, DEM, 340 students) and Graduate Entry Medical (GEM, 80 students) programs were invited to participate by means of a forum post (with attached Participant Information Leaflet) and to contact the principal investigator by e-mail if they wished to volunteer. All students who did so were assigned a unique participant number for pseudoanonymization by a gatekeeper, with no role or responsibility in teaching or assessing medical students. Within RCSI’s School of Medicine, the histology course was taught by self-directed online tutorials, within the first year of the curriculum, integrated into the systems-based, multidisciplinary modules, and students were advised to study the “Endocrine System” online histology tutorial prior to their interview (which was part of their normal course content for the semester) [35, 38].

### Preparation of Multiple-Choice Questions

Two examination papers with 14 multiple-choice questions were prepared by two content experts, with six identical anchor MCQs on each test (Table 1; Supplementary Information). Three anchor MCQs had textual vignettes only, and three items required interpretation of an adjacent image (“required image”). The remainder of MCQs on each test were matched, where one test had an MCQ with a textual vignette, and the matched MCQ on the other test included an image (Table 1; Supplementary Information). The image-based MCQ contained either (a) identical text along with an image containing information complementary but non-essential to answering the question (“redundant image”) or (b) a modified textual vignette, with removal of details critical to answering the question, and an image added to provide that required information or context (“required image”).

**Table 1:** Overview of assessment items completed by students in version A and version B of the tests in this study

			**vA**	**vB**
Q1	Anchor item	Diabetes insipidus	Textual vignette	(identical to vA)
Q2	Anchor item	Testes and Leydig cells	Text and required histological image	(identical to vA)
Q3	Matched item	Endoplasmic reticulum	Textual vignette	Identical text and redundant image (diagram)
Q4	Matched item	Placental human chorionic gonadotrophin (HCG)	Identical text and redundant histological image	Textual vignette
Q5	Matched item	Thyroid parafollicular cell function	Textual vignette	Modified text and required histological image
Q6	Matched item	Pancreatic islet of Langherhan’s function	Modified text and required histological image	Textual vignette
Q7	Anchor item	Pregnancy and progesterone	Textual vignette	(identical to vA)
Q8	Anchor item	Adrenal medulla and potassium dichromate	Text and required histological image	(identical to vA)
Q9	Matched item	Pituitary acidophils	Identical text and redundant histological image	Textual vignette
Q10	Matched item	Neuroendocrine cells and adrenal medulla	Textual vignette	Identical text and redundant image (diagram)
Q11	Matched item	Oxytocin secretion	Modified text and required image (diagram)	Textual vignette
Q12	Matched item	Adrenal cortex and androgens	Textual vignette	Modified text and required histological image
Q13	Anchor item	Holocrine secretion	Textual vignette	(identical to vA)
Q14	Anchor item	Pituitary gland	Text and required histological image	(identical to vA)

### Interviews

Students then met individually with one of the interviewers, and were given an opportunity to ask questions before giving formal consent, then randomly assigned to either version A or version B of the test (Table 1; Supplementary Information). Students completed demographic questions regarding their educational level and linguistic abilities (native and known languages), and then the Verbal–Visual Learning Style Rating (VVLSR; 7-point Likert), to identify whether they self-identified as predominantly verbal or visual learners [12]. Students were given some guidance on verbalizing their thoughts (“think-aloud”) and asked to answer two practice questions, voicing their thoughts as they completed these questions. Students then completed their 14 MCQs, while continuing to verbalize their thoughts for recording and transcription.

### Analyses

#### Quantitative Data

All demographic and test data were collated and tabulated in MS Excel, then imported to STATA 17.0 for statistical analysis (StataCorp., College Station, TX). A caveat must be stated that the primarily qualitative focus of this study, and the small number of participants (*n* = 30), limits the statistical power and thus the interpretation of quantitative statistical analyses. Differences were considered significant for values of *p* < 0.05 for all (parametric) statistical analyses performed in this study, with the mean and standard deviation used to summarize students’ scores. For analysis of the Verbal–Visual Learning Style Rating, the mean was again chosen as the measure of central tendency, and comparisons were performed by means of independent *t*-tests [39–43]. Item psychometrics were calculated in STATA, with item discrimination calculated by means of a point biserial correlation (pwcorr, a true Pearson product-moment correlation), with a higher positive correlation for an MCQ indicating that students who achieved a high score on the overall test also scored higher on that individual MCQ.

#### Qualitative Analysis

Transcripts were imported into QSR International’s NVIVO 11 qualitative data analysis software (QSR International Pty. Ltd.), which was used for all further thematic analyses. While some codes and themes were anticipated from prior reading of the existing evidence base, all transcripts were initially read for familiarity, then coded with a realist, inductive approach, with anticipated and additional emerging codes identified and integrated within themes [44–48]. The process was iterative, with the thematic structure undergoing revisions throughout, with coding and analysis shared and discussed between co-authors throughout the process [49]. Following development of the final thematic framework, a final formal analysis of 20% of scripts was performed by an additional coder for comparison with this final schema and themes.

## Results

### Quantitative Analyses and Item Statistics

All students completed the interview well within the 30 min allotted, with a mean recording length of 14 min and 5.5 s. Cronbach’s alpha (scale reliability coefficient) for version A of the test was 0.68, while version B was 0.67. Comparing students’ scores on the test papers overall, there was no statistically significant difference observed between students who completed version A of the test (*M* = 10, SD = 1.77) as compared to those who completed version B (*M* = 10.5, SD = 2.03; *t*(28) =  − 0.7663, *p* = 0.45). Item statistics for the six identical anchor MCQs showed no statistically significant difference in the item facility observed on version A of the test (*M* = 0.63, SD = 0.23) as compared to version B (*M* = 0.67, SD = 0.29; *t*(5) =  − 0.8076, *p* = 0.46). Similarly, no significant differences were observed in point-biserial correlation (0.266 ± 0.28 vs 0.384 ± 0.19; *t*(5) =  − 0.8611, *p* = 0.43) of these six anchor MCQs as answered by students completing either version A or B of the test.

For the remainder of the MCQs on the paper, these matched image MCQs either had (a) identical text and an additional (redundant) image that was not essential to answering the question or (b) modification of the vignette and substitution of textual information with an image (Table 1; Supplementary Information). Comparing the text-only MCQs with their match that had an identical textual vignette and an additional “redundant” image attached, a slight and non-significant reduction in item facility (0.75 vs 0.67; *p* = 0.08) was observed, but with no demonstrable impact on point-biserial correlation (0.43 vs 0.42; *p* = 0.96). Comparing text-only MCQs with their match containing modified text and an image requiring interpretation showed no statistically significant difference in either item facility (0.88 vs 0.88; *p* = 1.00) or point-biserial correlation (0.25 vs 0.20; *p* = 0.84).

There was no difference observed in the students’ self-identified VVLSR when comparing students who completed version A of the test (*M* = 4.67, SD = 1.5) with those who completed version B (*M* = 4.47, SD = 1.85; *t*(28) = 0.3259, *p* = 0.75). There was no significant difference between those who identified as more verbal, more visual, or equal learners with regard to overall score, or subscores on anchor MCQs, on text-only MCQs, or on MCQs with images overall (Table 2). For scores on the MCQs with redundant images, self-identified visual learners received a lower score on these two MCQs (1.1 ± 0.72) than either verbal (1.5 ± 0.53) or equal learners (1.67 ± 0.52), but this did not reach statistical significance (*F*_(2, 27)_ = 1.93; *p* = 0.165; Table 2).

**Table 2:** Scores and subscores on different item types by students with self-identified visual, verbal, or no learning preferences (VVLSR)

	**VVLSR – equal** **(*n* = 6)**	**VVLSR—more verbal (*n* = 8)**	**VVLSR – more visual (*n* = 16)**	***F***	*p* value*
**Participants’ test metrics and scores—mean (SD)**					
Overall score	11.17 (± SD 0.75)	10.5 (± SD 1.85)	9.81 (± SD 2.13)	(2, 27) 1.22	0.312
Anchor items (*n* = 6)	4.33 (± SD 0.52)	3.75 (± SD 1.49)	3.81 (± SD 1.04)	(2, 27) 0.58	0.576
Items – text (*n* = 7)	6 (± SD 0.63)	5 (± SD 1.07)	4.94 (± SD 1.24)	(2, 27) 2.14	0.137
Items – image (*n* = 7)	5.17 (± SD 1.17)	5.5 (± SD 1.2)	4.88 (± SD 1.36)	(2, 27) 0.64	0.535
* Image required (n* = *5)*	*3.5 (*± *SD 1.05)*	*4 (*± *SD 1.07)*	*3.75 (*± *SD 0.93)*	*(2, 27)* *0.44*	*0.648*
* Image redundant (n* = *2)*	*1.67 (*± *SD 0.52)*	*1.5 (*± *SD 0.53)*	*1.1 (*± *SD 0.72)*	*(2, 27)* *1.93*	*0.165*

^*^One-way ANOVA

### Qualitative Exploration of Cognitive Processes

All 30 students each verbalized their responses to 14 questions, resulting in 420 student-item verbalizations. The verbalizations and cognitive processes observed are organized under three main themes within which sub-themes were developed (Table 3). The first theme concerned non-inferential description of the students’ vocalizations or observed behaviors, including sub-themes of reading the vignette fully (verbatim), linguistic, or language mispronunciations, admitting knowledge deficits, or returning to review or reread MCQs prior to completing the paper [48]. The second theme involved identification of reasoning or cognitive strategies that students used to answer the question, incorporating sub-themes including generating a correct answer from ready knowledge before reviewing options, using option elimination to select an answer, or selecting an incorrect option with no obvious verbalization or consideration of the correct option (premature closure) [34, 48]. Another sub-theme was whether students noticed and used the deliberate vertical cues that were inserted into the papers, whereby information in one MCQ aided in answering another MCQ on the paper. The third theme included all observed verbalizations and inferred cognitive processes specifically related to image identification or analysis: analytical, non-analytical, and image not mentioned [46, 47]. Analytical observations made specific reference to features such as scale, shape, or color to deduce the answer. Non-analytical observations gave no indication as to how (named) features were identified, and for many of the image-based MCQs, there were simply no verbalizations related to the image at all.

**Table 3:** Themes and sub-themes with indicative examples of recorded verbalizations

	Definition	Total count*	Examples
**Observed vocalizations and behaviors** *(14* × *30* = *420 student-item verbalizations)*			
Reading vignette fully	Reading the vignette verbatim	403 / 420	Question 2, what is produced by the cells indicated by the arrow in the image to the right? ^P14^
Language—minor misspeaking or mispronunciation	Mispronunciation—does not infer conceptual misunderstanding	20 / 420	I think they’re the *ledwig [sic]* cells. So I’m going to pick E – testosterone there. ^P21^ Right, so you have your GFR. So you have, your reticularis, your fascicularis [sic] and your glomerulus [sic].^P23^
Systematically reading or listing all options aloud	Listing all options systematically	59 / 420	… the condition is typically caused by disease or damage to which of the following structures? A – adrenal medullary, B – anterior pituitary, C – hypothalamus, D – pancreas, E – renal cortex. ^P14^
Returned and reviewed items and answers	Returned to reconsider or check an item answer for a second (or third) time before completing test	153 / 420	Okay, I’ll check them one last time. ^P10^ So there was two then, two or three I wanted to go back over and none which had questions later on in the test that helped. ^P11^
Admitting knowledge deficits	Explicitly expressing unfamiliarity or lack of knowledge	85 / 420	Potassium dichromate, dichromate was made, like something salt based or whatever it’s called. Em, then I don’t know, I don’t know. ^P10^ I just can’t think of what cells they are. (Short pause). Oh maybe (short pause) because all the options don’t seem familiar. ^P16^
**Reasoning or cognitive strategies** *(14* × *30* = *420 student-item verbalizations)*			
Generates (correct) answer before reviewing options	Upon reading the vignette the participant immediately generates the correct answer, *before* reading the option list	194 / 420	Question two, what is produced by cells, the cells indicated by the following arrow so they’re, em, the testis (laughs) and the cells in between are leydig cells or interstitial cells and they produce testosterone. ^P13^ While attending the obstetric clinic, you see and examine a number of ladies attending their routine pre-natal visit. Which of the following hormones is produced by the placenta during pregnancy? (Sighs) I’m going to go with HCG. ^P22^
Eliminating options—no initial self-generation	No initial correct answer is verbalized, and the participant reads through the option list, systematically eliminating options to arrive at an answer	151 / 420	Endocrine cells manufacture hormones, which are then exported to act on receptors at distant sites in the body. Which of the following organelles plays a significant role in the manufacturing of hormones within the cell? It’s not the mitochondria. It’s not vesicles is what exports it, em, (short pause); endoplasmic reticulum should put it all together. They come together. Packages are the vesicles. Nucleus is the site is the site of gene expression so it should be the Golgi apparatus. ^P19^
Reaching (incorrect) closure prematurely	Failure to consider the correct alternative in answering the test item	43 / 420	Within the endocrine system, what is the mode of secretion in which the cell membrane ruptures and the entire contents of the cells are shed? So membrane is the apocrine, apocrine. ^P7^
Reaching closure with difficulty or delay	(1) Pattern of thinking failing to help answer the question, (2) excessive length of time and commentary to answer the question, (3) excessive pauses, and (4) taking tangents	53 / 420	*Word count prohibitive to include concise examples*
Noticing a vertical cue *(inserted intentionally!)*	Using information provided in one item to assist in answering another item on the paper	8 / 420	Okay, I won’t go for thyroid. Pancreas we’ve seen pictures of it before, so it’s not that. Adrenal, okay, I’ll guess pituitary because I don’t really know. (Turns pages). ^P27^ This is where I remember that on a previous page, neuroendocrine derivative cells have been mentioned, so I’ll go back and (turns page) and check and question eight says the polyhedral cells… ^P30^
**Image identification and / or analysis** *(7* × *30* = *210 student-item verbalizations)*			
Analytical observations	Verbalizations regarding scale, size, shape	108 / 210	That’s two millimetres so it’s probably, what gland is that is it the parathyroid or the pituitary. I’m going to say the pituitary gland because it look like it’s a very tiny gland but I’m not… ^P15^ …Em, I think it’s either eosin or haematoxylin because they look kind of blue and pink, so eosin is the pink one. They look kind of pink. Because I wrote my notes in pink for eosin. ^P16^
Non-analytical observations	The tissue or a structure is named, but with no indication as to how it was recognized or interpreted	44 / 210	… What is produced by the cells indicated by the arrow in the image to the right? It’s the leydig cells in the testes so its testosterone. ^P7^ Question twelve, the adrenal cortex has three distinguishable zones that secrete steroid hormones. Which of the following hormones are secreted by the layer of the cortex labelled “B” in the image on the right? B is zona reticularis which produces sex hormones so its androgens I think. ^P16^
Image not mentioned	No verbalization about the image	58 / 210	*No verbalizations to include as examples*
Redundant image	*(2* × *30* = *60 maximum)*	50 / 60	*No verbalizations to include as examples*
Required image	*(5* × *30* = *150 maximum)*	8 / 150	*No verbalizations to include as examples*

^*^Referring to the number of student-item verbalizations in which this code was observed

### Student Performance

Most students read the vignette and question aloud, fully, and verbatim (403 of 420 student-item verbalizations; Tables 3, 4), but the list of options was seldom read in the same systematic manner (59 of 420 student-item verbalizations). High-performing students were significantly more likely to self-generate an immediate answer to MCQs, without any verbalization indicating that they had read the full option list (74 of 126 student-item verbalizations; 59%), than medium (94 of 210; 44.8%) or lower (26 of 84; 31%) performing students (*F*_(2, 27)_ = 6.60, *p* = 0.0046; Tables 3, 4). Not all apparently self-generated answers were correct. There were 43 verbalizations where a student reached (incorrect) closure prematurely, generating an answer by selecting an incorrect option, without any verbal indication that the correct option had been read or considered (Table 3). Premature closure was observed more frequently in verbalizations from lower performing students (16 of 84 student-item verbalizations; 19.1%) as compared to medium (23 of 210; 11%) or high (4 of 126; 3.2%) performing students (*F*_(2, 27)_ = 7.33, *p* = 0.0029; Table 4).

**Table 4:** Comparison of verbalizations from high, medium, and lower performing students

	**Count (percentage) of test items demonstrating a behavior or cognitive strategy according to student performance**			*p* value*
	High performer (score ≥ 12; *n* = 9)	Medium performer (score 9 – 11; *n* = 15)	Low performer (score ≤ 8; *n* = 6)	
**Observed vocalizations and behaviors**				
Reading vignette fully	122 / 126 (96.8%)	198 / 210 (94.3%)	83 / 84 (98.8%)	0.4724
Language—minor misspeaking or mispronunciation	1 / 126 (0.8%)	16 / 210 (7.6%)	3 / 84 (3.6%)	0.1192
Systematically reading or listing all options aloud	19 / 126 (15.1%)	30 / 210 (14.3%)	10 / 84 (11.9%)	0.9655
Returned and reviewed answers	17 / 126 (13.5%)	78 / 210 (37.1%)	58 / 84 (69.1%)	0.0577
Admitting knowledge deficits	9 / 126 (7.1%)	49 / 210 (23.3%)	27 / 84 (32.1%)	**0.0114**
**Reasoning or cognitive strategies**				
Generates (correct) answer before reviewing options	74 / 126 (58.7%)	94 / 210 (44.8%)	26 / 84 (31%)	**0.0046**
* No verbalization of reviewing alternative options*	*55 / 126 (43.7%)*	*71 / 210 (33.8%)*	*18 / 84 (21.4%)*	
* Checks options after generating (correct) answer*	*19 / 126 (15.1%)*	*23 / 210 (11%)*	*8 / 84 (9.5%)*	
Eliminating options—no initial self-generation	38 / 126 (30.2%)	78 / 210 (37.1%)	35 / 84 (41.7%)	0.3997
Reaching (incorrect) closure prematurely	4 / 126 (3.2%)	23 / 210 (11%)	16 / 84 (19.1%)	**0.0029**
Reaching closure with difficulty or delay	1 / 126 (0.8%)	30 / 210 (14.3%)	22 / 84 (26.2%)	**0.0034**
Noticing a vertical cue	1 / 126 (0.8%)	7 / 210 (3.3%)	0.00 (00.0%)	0.3515
**Image interpretation *(image items n* = *7)***				
Analytical observations (scale, size, shape, color etc.)	31 / 63 (49.2%)	58 / 105 (55.2%)	19 / 42 (45.2%)	*p* = 0.612**
Non-analytical observations	12 / 63 (19.1%)	20 / 105 (19.1%)	12 / 42 (28.6%)	
Image not mentioned	20 / 63 (31.8%)	27 / 105 (25.7%)	11 / 42 (26.2%)	
* Redundant image (n* = *2)*	*14 / 18 (77.8%)*	*25 / 30 (83.3%)*	*11 / 12 (91.7%)*	
* Required image (n* = *5)*	*6 / 45 (13.3%)*	*2 / 75 (2.7%)*	*0 / 75 (0%)*	

^*^One-way ANOVA between high-medium–low–performing groups

^**^X^2^(4) = 2.6818, *p* = 0.612

Unsurprisingly, lower performing students were more likely to verbalize about knowledge deficits, or being uncertain, than medium- or high-performing students (*F*_(2, 27)_ = 5.31, *p* = 0.0114; Tables 3, 4). Lower performing students also appeared to have more difficulty or delay in answering MCQs (22 of 84 student-item verbalizations; 26.2%), compared to medium (30 of 210; 14.3%) or high (1 of 126; 0.8%) performing students (*F*_(2, 27)_ = 7.05, *p* = 0.0034; Tables 3, 4). Lower performing students were also more likely to return and review MCQs for a second, or even a third time, before completing the test (*F*_(2, 27)_ = 3.18, *p* = 0.0577; Tables 3, 4). This study was designed to include a small number of vertical cues on each paper, but few students appeared to notice these, as they were remarked upon in only eight verbalizations (three of which were from one individual student (Tables 3, 4)).

#### Image Interpretation

Students verbalized more observations when answering MCQs containing an image which was necessary or essential to answering the question, whereas redundant images were unlikely to be mentioned by students at all (*X*^2^(2) = 133.0720, *p* < 0.001; Fisher’s exact test, *p* < 0.001; Table 5). Students who self-identified as verbal learners were more likely to have a verbally analytical approach to answering MCQs with images, making specific comments about the scale, shape, features, or colors within the image (*X*^2^(4) = 17.8040, *p* = 0.001; Fisher’s exact test, *p* = 0.001; Table 6).

Right it’s not methylene blue because I don’t see any blue indications in the image. ^P01^

So, I know that the predominant stain for an awful lot of the slides was the H&E one and what I’m looking at doesn’t look as pink as some of those. I’m going to scratch out A which is eosin and B which is haematoxylin. Definitely not methylene blue because they look kind of red. ^P11^

Okay, I have to analyse this image because there is no colloid, so I don’t think it will be thyroid. No follicles evident. It does look like it has two lobes though so it could be pituitary. It’s probably pancreas, no I don’t think it would be pancreas. It has no follicles as well. I don’t think it would be adrenal either, just because split in two I’ll go with pituitary. ^P12^

Em, okay, this looks like its pointing at a thing in between the big things, so I’m going to guess that it’s interstitial cell or a leydig cell, so testosterone is what I will choose. ^P21^

Well it looks like there is two distinct stains, one is lighter than the other, em, and one’s bigger than the other. So it looks like an anterior and a posterior pituitary to me, so I’m going to say pituitary.^P29^

**Table 5:** Comparison of verbalizations from students when answering image-based MCQs with either a “redundant image” or a “required image”

	**Count (percentage) of verbalizations, according to image type**		
	*Redundant image (n* = *2)*	*Required image (n* = *5)*	
Analytical observations (scale, size, shape, color, etc.)	3 / 60 (5%)	105 / 150 (70%)	*p* < 0.001*
Non-analytical observations	7 / 60 (11.7%)	37 / 150 (24.7%)	
Image not mentioned	50 / 60 (83.3%)	8 / 150 (5.3%)	

^*^X^2^(2) = 133.0720, *p* < 0.001; Fisher’s exact test, *p* < 0.001

**Table 6:** Comparison of verbalizations according to students’ self-identified Verbal–Visual Learning Style Rating (VVLSR) when answering image-based MCQs

	**Number of student-item verbalizations demonstrating a particular behavior or cognitive strategy according to self-identified VVLSR**			
	Visual learners (*n* = 16)	No preference (*n* = 6)	Verbal learners (n = 8)	
**Image identification and / or analysis** *(7* × *30* = *210 student-item verbalizations)*				
Analytical	45 / 112 *(40.2%)*	24 / 42 *(57.1%)*	39 / 56 *(69.6%)*	*p* = 0.001*
Non-analytical	31 / 112 *(27.7%)*	10 / 42 *(23.8%)*	3 / 56 *(5.4%)*	
Image not mentioned	36 / 112 *(32.1%)*	8 / 42 *(19%)*	14 / 56 *(25%)*	
* Redundant image* * (2 per participant)*	*30 / 32 (93.8%)*	*7 / 12 (58.3%)*	*13 / 16 (81.3%)*	
* Required image* * (5 per participant)*	*6 / 80 (7.5%)*	*1 / 30 (3.3%)*	*1 / 40 (2.5%)*	

^*^X^2^ (4) = 17.8040, *p* = 0.001; Fisher’s exact test, *p* = 0.001

Visual learners were more likely to make non-analytical comments about the image, where students would mention the image, perhaps even naming a structure seen within it, but giving no verbal indication as to how they had identified, interpreted, or analyzed it.

I’m going to go with testosterone because I feel like they look like leydig cells. ^P01^

so the arrow’s pointing the posterior pituitary which makes oxytocin and ADH, so the only answer is oxytocin. ^P13^

Em, there’s a picture as well, em, so I suppose the picture is just to remind you ^P17^

So I know that this is going to be the glomerulus, this is the glom, this is the fasculata and this is reticularis so that’s going to be your androgens. ^P18^

…for this question I didn’t really use the image on the right since it wasn’t really useful to me, since it didn’t relate to the actual thought process. ^P30^

## Discussion

This study sought to explore (1) the cognitive processes and critical thinking of students while answering single best answer MCQs in histology, (2) whether images influence the verbalized cognitive processes of students, and (3) whether self-identified verbal and visual learners display different verbalizations or cognitive processes when answering text and image-based MCQs. The “think-aloud” method explores metacognition through the lens of viewing thinking as inner speech, where people externally vocalize their inner monologue, and is accepted as a valid research methodology to explore reasoning and problem-solving in many fields, including medicine [34, 47, 48, 50–52]. There are some criticisms, such as the potential for this ongoing verbalization to cause people to use limited cognitive resources on incidental processing, leaving less cognitive capacity for essential processing, or to potentially interrupt or influence the internal voice [9, 53, 54].

Another potential issue is that not all thinking or cognition is performed in an analytical manner, subject to being easily verbalized. Intuitive leaps, unconscious biases, subconscious pattern recognition—these subconscious thoughts will *not* be captured by verbalization of an inner monologue, although they may still heavily influence decision making, particularly when addressing complex questions or contexts [53, 55–58]. The finding that high-performing students were significantly more likely to self-generate an answer as compared to middle and lower performing students is consistent with observations in related studies of reasoning [46, 48, 59] and is the theoretical basis for the development and use of the very-short-answer question format [60]. Lower performers not only were more likely to verbalize about knowledge deficits, but also more likely went back to check or change answers than other students, which is also a finding consistent with previous studies [48].

Image recognition and interpretation are key skills in many of the medical sciences [31, 34, 61–65]. Therefore, the principles of constructive alignment mean that visual interpretation and analysis should be an integral, albeit proportional, part of assessment strategy and design [32]. While few assessments may specifically assess these skills of visual interpretation and analysis, those that do are typically well received by students, who appreciate their authenticity in preparing them for clinical practice, sentiments mirrored by students in this study [11, 66, 67]. While much prior research has reported that inserting images to a single best answer MCQ has no influence overall item psychometrics per se [35, 68–71], other studies have reported inconsistent effects and hypothesized that these effects are due to the qualities or characteristics of the image used [24]. Other studies have contrasting findings, reporting that students’ scores are higher when answering MCQs with images [67], or conversely that the inclusion of images *reduces* item facility (the inverse of item difficulty), lowering scores, potentially due to increasing extraneous cognitive load or *spatial contiguity* effects [18, 20].

Despite the small number of MCQs with redundant images in this study, the manner in which these images went mostly unmentioned in students’ verbalizations, along with the reduced item facility for these MCQs, strongly suggests that redundant images are a hindrance in assessments, not a help, and should not be included within MCQ vignettes. While no other comparable research has been done to date within medical assessment, the inclusion of “irrelevant, redundant or interacting sources of information” in arithmetic examinations is also suggested to slow down the speed at which students are able to process information, leading to increased testing time and item difficulty (the inverse of item facility) [37]. This *coherence effect* strongly suggests that over-excessive detail reduces capacity for essential information processing, and thus potentially detrimental to students’ performance in assessments [9, 19, 72, 73].

However, this study also demonstrated that the inclusion of an image that *was* essential to correctly answering an MCQ did not appear to have any significant influence on item psychometrics or observed verbalizations as compared to text-alone vignettes. Thus, the use of images in MCQ vignettes written to specifically test the ability of candidates’ ability to identify or interpret *required* images not only is no threat to validity, but also is logically required according to the principles of constructive alignment [27, 32, 68–71]. Furthermore, students’ self-identified VVLSR had no discernable influence on their objective scores when answering verbal or visual MCQs. Verbal learners were significantly more analytical in their verbalizations when answering image-based MCQs than visual learners, but learners who self-identify as being verbal learners may de facto experience a more analytical inner verbal monologue than those who self-identify as visual learners [12, 16, 17, 58].

Additional factors, such as the quality of the images provided for candidates, along with their spatial (or temporal) relationship to the placement of the question text, do merit some conscious consideration when writing MCQs [9, 19, 20, 27, 33, 34, 74]. Where text and image are spatially separated on separate sheets or screens, some processing capacity will be diverted from image interpretation by the necessity to switch visual focus, looking back and forth between text and image [19, 20, 73]. Where this is not possible, and the image and text are separated, research on cognition suggests text should be placed to precede the image to provide context, with a caveat that this is as yet not definitively researched in MCQ assessments [19, 33].

The hypothesis that the characteristics or complexity of the images used in MCQ vignettes will affect item statistics and metacognition has been recognized by numerous authors [11, 19, 24, 34, 75]. Sagoo et al. found that students scored significantly higher on questions with images (both anatomical and radiological) compared to questions without images [11]. Further analysis considering image subtypes demonstrated that “students performed significantly better on questions referring to bones than to soft tissues regardless of the image type [anatomical or radiological]” suggesting that visual interpretation of an isolated structure (a bone) is less complex than the synthesis of information required to interpret images of interrelated and intersecting soft tissues [11]. The simplicity or complexity of both verbal vignettes and images may be accounted for within assessment strategies or processes, for example mapping to cognitive taxonomies [23, 76–78]. Analysis of histological and cross-sectional images requires interpreting complex “categorical spatial relations,” whereby the relationships between objects must be judged [34, 79]. Students seem to struggle more with MCQs displaying cross-sectional illustrations, as compared to those which use simpler diagrams or line drawings [24], and to demonstrate different cognitive processes when answering MCQs nested within cross-sectional themes, with more reliance on option elimination, and less visualizing or verbal reasoning being described [34]. It is essential for students to consciously develop and improve these visual and spatial interpretive skills by training and practice, as opposed to passively noting what is pointed out to them, or only memorizing a limited set of exemplars, so that they can apply their knowledge when viewing unfamiliar or novel images, whether in assessments or in future independent practice [14, 19, 75, 79, 80]. For this reason, perhaps a number of novel images should be used within assessments if the required aim is to truly test the students’ abilities of image interpretation, even aside from the argument that the use of familiar images may promote positive cueing [24, 34, 36], an item flaw that may also be present in purely textual vignettes [81]

Interpretation of images and spatial relationships is essential in many disciplines and so including images within assessments aids authenticity and constructive alignment. While the effects of visual or multimedia learning have been explored in many contexts, guidelines for assessment are still sparse, but some basic principles can be considered. Firstly, is the image *relevant and essential* to answering the question? Redundant information, including images, may simply increase extraneous cognitive load to no benefit, potentially influencing student performance [19, 37, 72, 80]. Secondly, does the image show the relevant structure in isolation, such as an individual bone, or is it seen *in relation* to surrounding structures, as is the case with a histological cross section, or an abdominal CT scan? Interpreting spatial relations is certainly appropriate for many assessments, but increases the difficulty of the task or question. The third point of *recognition* considers whether the candidate is presented with a familiar image, seen and studied during their learning activities, or an entirely unfamiliar one. Novel images can be true tests of a candidate’s ability to demonstrate their knowledge and skill at image interpretation, but is potentially more cognitively demanding than recognizing a familiar image, or at least one similar to previous images studied. The *realism* of the image could also be considered; is the image a simple diagram, or is it a photograph of an actual histological or anatomical specimen [82]? Finally, for formatting, the *spatial contiguity* principle states that images should be as close as possible to their corresponding text, so that they may be viewed simultaneously without a need to switch focus, as opposed to being on a separate page or screen [19, 33].

### Limitations of the Study

This research study was designed as a qualitative think-aloud exploration of cognitive processes. While quantitative statistical analyses were performed and reported, the small number of participants (*n* = 30) limits the statistical power of these quantitative analyses. Furthermore, the cohort of students who participated were all volunteers, and so entirely self-selecting. While it was conducted in one institution, RCSI encompasses highly diverse student and staff bodies, and in this study of 30 students only 11 recorded their nationality as being within the EU, and students from Asia, North America, and the Middle East were all represented. Only 12 students were monolingual English speakers, 15 identified as bilingual, with two students speaking three languages fluently and one individual fully confident in four. However, the authors’ hope that the findings of this study will stimulate further interest and provide some supporting evidence for future investigation in this field.

## Conclusions

In summary, high-performing students were significantly more likely to self-generate an answer as compared to middle and lower performing students, who relied to a greater degree on option elimination. Adding images to MCQs did not have a consistent influence on item statistics, and the students’ self-identified visual-verbal preference (“learning style”) had no consistent bearing on their results for text or image-based questions. Students’ verbalizations regarding images are very highly dependent on whether the image was necessary or unnecessary to answering the question. For MCQs where interpretation of the image was required, specific references to the image were noted for 95% of student-item verbalizations (142 of 150 maximum). In contrast, for MCQs where the image was redundant or unnecessary to answering the MCQ, reference to the image was recorded in only 17% of student-item verbalizations (10 of 60 maximum). The finding does align with the principles of question writing, whereby MCQ vignettes should not be cluttered with unnecessary information that do not help with cueing or answering MCQs, and may instead be detrimental distractions, adding to extraneous cognitive load.

## Supplementary Information

Below is the link to the electronic supplementary material.

Supplementary file1 (DOCX 1315 KB)

## Declarations

### Supplementary Information

Interview sheets & MCQs.
