# Supplementary material for: A Qualitative Exploration of Student Cognition When Answering Text-Only or Image-Based Histology Multiple-Choice Questions
Source: Med Sci Educ. 2024 Jul 24;34(6):1317–29. doi: 10.1007/s40670-024-02104-x (PMC11699003; doi:10.1007/s40670-024-02104-x)
Supplement: Supplementary file 1 — Supplementary file1 (DOCX 1315 KB) [file 40670_2024_2104_MOESM1_ESM.docx]

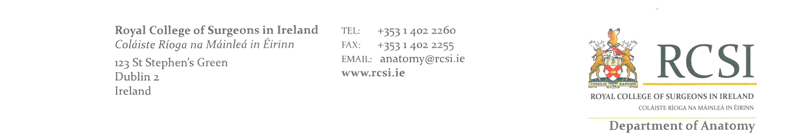


A

MCQs & Images:

*Think-aloud analysis of cognitive processes and test-taking strategies, comparing illustrated vs. text-alone vignettes in histology multiple choice questions.*

Thank you again for agreeing to part in this study. Both this answer sheet and your audio recording (and subsequent transcript) will be anonymised, so we would be grateful if you could indicate some basic demographic information for us:

1. Have you previously completed an undergraduate degree (i.e. B.Sc., Nursing)?

Yes 🗌 No 🗌

2. Which program are you enrolled in?

5/6 year Medicine 🗌 Graduate Entry Medicine 🗌

3. How many languages are you fluent in?

4. Please indicate the option which best describes your nationality:

EU 🗌 N. America 🗌 Middle East 🗌 Asia 🗌 Other 🗌

5. What is your native language?

English 🗌 Arabic 🗌 Malay 🗌 Chinese 🗌 Other 🗌

6. VVLSR – (Verbal-Visual learning preference)

| In a learning situation sometimes information is presented verbally (e.g. with printed or spoken words) and sometimes information is presented visually (e.g. with labelled illustrations, graphs, or narrated animations). Please place a check mark indicating your learning preference. | | | | | | |
| --- | --- | --- | --- | --- | --- | --- |
| 🞅 | 🞅 | 🞅 | 🞅 | 🞅 | 🞅 | 🞅 |
| Strongly more verbal than visual | Moderately more verbal than visual | Slightly more verbal than visual | Equally verbal and visual | Slightly more visual than verbal | Moderately more visual than verbal | Strongly more visual than verbal |

7. Please indicate if there is any other information you feel may be of relevance to the research team (i.e. colour-blindness, synaesthesia) – alternatively, leave blank.

__________________________________

The interviewer will now briefly remind you how to “think-aloud” while answering questions during this interview. The purpose of this study is to explore how you think and reason, when answering MCQs. Here are two initial questions to practice “thinking aloud”, before we start the actual interview questions and the recording device:

Type I diabetes is characterised by a lack of insulin production. Which of the following glands produces insulin?

1. Adrenal
2. Pancreas
3. Parotid
4. Pituitary
5. Thyroid


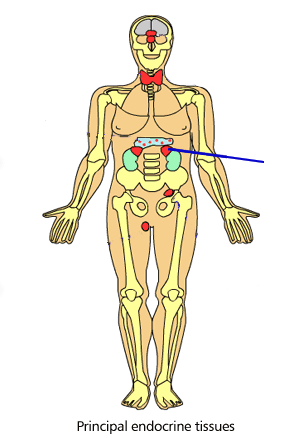


There are many endocrine cells and glands within the body, producing hormones. Which of the following structures is indicated by the blue line in the diagram to the right?

1. Adrenal Gland
2. Ovary
3. Pituitary gland
4. Testis
5. Uterus

The first thing that you will be asked to do when the recording starts is to state the number / version of the paper that you are answering – this will be your identifying number throughout the study from now on (you will be given this number again before you leave the interview for your own information).

**Recording starts:**

Please state your participant number aloud:

**A___**

Now turn over the page and answer the questions, while verbalising your thoughts (thinking aloud):

Q1. Diabetes insipidus is a condition in which the urine cannot be concentrated, so huge amounts of water are excreted, and the person must drink copiously to compensate. This condition is typically caused by disease or damage to which of the following structures?

1. Adrenal medulla
2. Anterior pituitary
3. Hypothalamus
4. Pancreas
5. Renal cortex


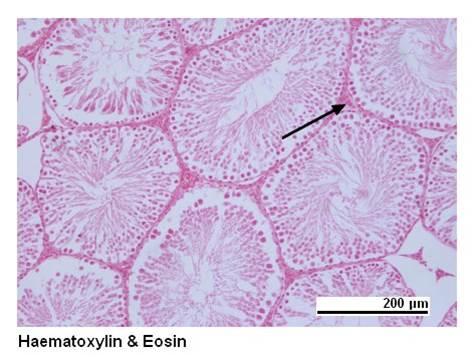
Q2. What is produced by the cells indicated by the arrow in the image to the right?

1. Alkaline phosphatase
2. Aldosterone
3. Insulin
4. Oestrogen
5. Testosterone

Q3. Endocrine cells manufacture hormones, which are then exported to act on receptors at distant sites in the body. Which of the following organelles plays a significant role in the manufacturing of these hormones within the cell?

1. Endoplasmic reticulum
2. Golgi apparatus
3. Mitochondria
4. Nucleus
5. Vesicles


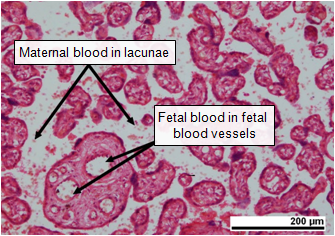
Q4. While attending the obstetric clinic, you see and examine a number of ladies attending for their routine pre-natal visit. Which of the following hormones is produced by the placenta during pregnancy?

1. Follicle stimulating hormone (FSH)
2. Growth hormone
3. Human Chorionic Gonadotrophin (HCG)
4. Luteinising Hormone (LH)
5. Prolactin

Q5. The thyroid gland contains multiple closed cavities surrounded by follicular cells, which produce T3 & T4. In contrast, parafollicular cells (C cells) are found between follicles or at the edge of a follicle.  What do these produce?

1. Calcitonin
2. Dopamine
3. Hydrocortisone
4. Oxytocin
5. Progesterone


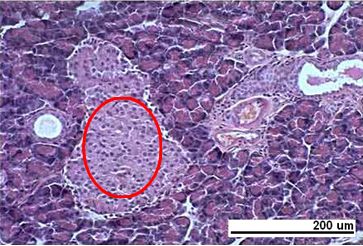
Q6. Which of the following cells is the predominant (most common) type to be found in area circled in the image to the right?

1. A - producing glucagon
2. B - producing Insulin
3. D - producing somatostatin
4. F - producing VIP
5. PP - producing substance P

Q7. A 26-year-old lady visits her Doctor for a routine pre-natal visit. If her serum hormones were to be tested, her level of progesterone would be seen to be much higher than in a non-pregnant woman. Where is this progesterone being produced?

1. Adrenal cortex
2. Anterior pituitary
3. Hypothalamus
4. Kidney
5. Ovary


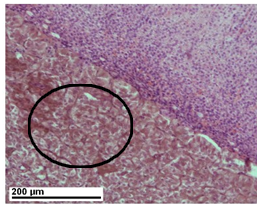


Q8. The polyhedral cells of the adrenal medulla are derived from neuroendocrine cells, and produce amines, like adrenaline and noradrenaline. What histological dye has been used to stain these polyhedral cells (circled) in the image to the right?

1. Eosin
2. Haematoxylin
3. Methylene blue
4. Potassium dichromate
5. Van Gieson’s


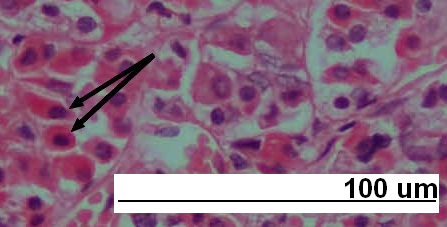
Q9. Chromophil cells within the pituitary are so named because they have a good affinity for the colours in certain stains. In sections stained with haematoxylin and eosin, what name is given to the chromophil cells which take up the pink dye?

1. Acidophils
2. Argentaffin cells
3. Basophils
4. Neutrophils
5. Chromaffin cells

Q10. Multiple endocrine neoplasia is a rare condition that effects endocrine cells derived from the neural crest. Which of the following cells are derived from neuroendocrine cells and so may be involved?

1. Adrenal medulla cells
2. Hepatocytes
3. Juxtaglomerular cells
4. Leydig cells
5. Thyroid follicular cells


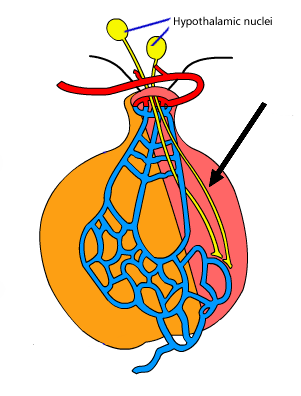
Q11. The pituitary gland forms a functional unit with the hypothalamus and has two principal parts, the anterior and posterior pituitary. Which of the following hormones is secreted by the part indicated by the arrow in the image to the right?

1. Aldosterone
2. Dopamine
3. Growth hormone
4. Oxytocin
5. Prolactin

Q12. The adrenal cortex has three distinguishable zones that secrete steroid hormones. Which of the following hormones are secreted by the innermost layer of the cortex?

1. Androgens
2. Catecholamines
3. Glucocorticoids
4. Gonadotrophins
5. Mineralocorticoids

Q13. Within the endocrine system, what is the mode of secretion in which the cell membrane ruptures and the entire contents of the cell are shed?

1. Apocrine
2. Autocrine
3. Holocrine
4. Merocrine
5. Paracrine


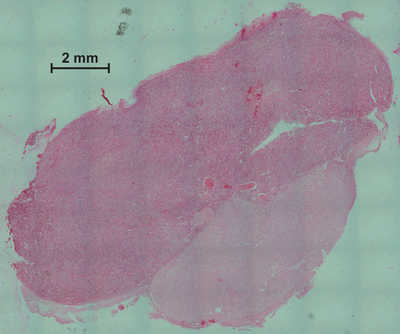
Q14. The image to the right shows shows which of the following glands?

1. Adrenal
2. Pancreas
3. Parathyroid
4. Thyroid
5. Pituitary

**End of Questions**


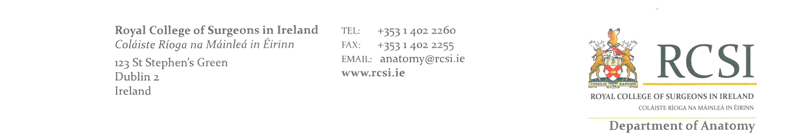


B

MCQs & Images:

*Think-aloud analysis of cognitive processes and test-taking strategies, comparing illustrated vs. text-alone vignettes in histology multiple choice questions.*

Thank you again for agreeing to part in this study. Both this answer sheet and your audio recording (and subsequent transcript) will be anonymised, so we would be grateful if you could indicate some basic demographic information for us:

1. Have you previously completed an undergraduate degree (i.e. B.Sc., Nursing)?

Yes 🗌 No 🗌

2. Which program are you enrolled in?

5/6 year Medicine 🗌 Graduate Entry Medicine 🗌

3. How many languages are you fluent in?

4. Please indicate the option which best describes your nationality:

EU 🗌 N. America 🗌 Middle East 🗌 Asia 🗌 Other 🗌

5. What is your native language?

English 🗌 Arabic 🗌 Malay 🗌 Chinese 🗌 Other 🗌

6. VVLSR – (Verbal-Visual learning preference)

| In a learning situation sometimes information is presented verbally (e.g. with printed or spoken words) and sometimes information is presented visually (e.g. with labelled illustrations, graphs, or narrated animations). Please place a check mark indicating your learning preference. | | | | | | |
| --- | --- | --- | --- | --- | --- | --- |
| 🞅 | 🞅 | 🞅 | 🞅 | 🞅 | 🞅 | 🞅 |
| Strongly more verbal than visual | Moderately more verbal than visual | Slightly more verbal than visual | Equally verbal and visual | Slightly more visual than verbal | Moderately more visual than verbal | Strongly more visual than verbal |

7. Please indicate if there is any other information you feel may be of relevance to the research team (i.e. colour-blindness, synaesthesia) – alternatively, leave blank.

__________________________________

The interviewer will now briefly remind you how to “think-aloud” while answering questions during this interview. The purpose of this study is to explore how you think and reason, when answering MCQs. Here are two initial questions to practice “thinking aloud”, before we start the actual interview questions and the recording device:

Type I diabetes is characterised by a lack of insulin production. Which of the following glands produces insulin?

1. Adrenal
2. Pancreas
3. Parotid
4. Pituitary
5. Thyroid


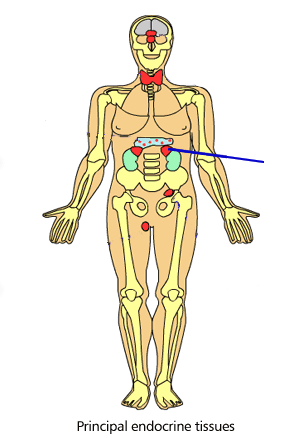


There are many endocrine cells and glands within the body, producing hormones. Which of the following structures is indicated by the blue line in the diagram to the right?

1. Adrenal Gland
2. Ovary
3. Pituitary gland
4. Testis
5. Uterus

The first thing that you will be asked to do when the recording starts is to state the number / version of the paper that you are answering – this will be your identifying number throughout the study from now on (you will be given this number again before you leave the interview for your own information).

**Recording starts:**

Please state your participant number aloud:

**B___**

Now turn over the page and answer the questions, while verbalising your thoughts (thinking aloud):

Q1. Diabetes insipidus is a condition in which the urine cannot be concentrated, so huge amounts of water are excreted, and the person must drink copiously to compensate. This condition is typically caused by disease or damage to which of the following structures?

1. Adrenal medulla
2. Anterior pituitary
3. Hypothalamus
4. Pancreas
5. Renal cortex


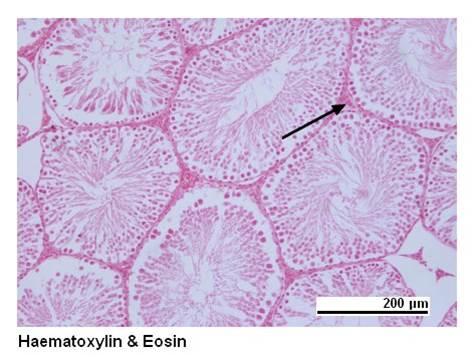
Q2. What is produced by the cells indicated by the arrow in the image to the right?

1. Alkaline phosphatase
2. Aldosterone
3. Insulin
4. Oestrogen
5. Testosterone


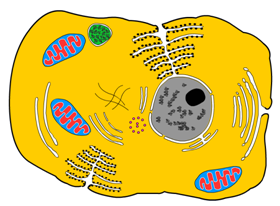
Q3. Endocrine cells manufacture hormones, which are then exported to act on receptors at distant sites in the body. Which of the following organelles plays a significant role in the manufacturing of these hormones within the cell?

1. Endoplasmic reticulum
2. Golgi apparatus
3. Mitochondria
4. Nucleus
5. Vesicles

Q4. While attending the obstetric clinic, you see and examine a number of ladies attending for their routine pre-natal visit. Which of the following hormones is produced by the placenta during pregnancy?

1. Follicle stimulating hormone (FSH)
2. Growth hormone
3. Human Chorionic Gonadotrophin (HCG)
4. Luteinising Hormone (LH)
5. Prolactin


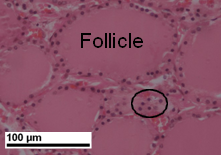
Q5. The thyroid gland contains multiple closed cavities surrounded by follicular cells, which produce T3 & T4. In contrast, what is produced by the circled cells in the image to the right?

1. Calcitonin
2. Dopamine
3. Hydrocortisone
4. Oxytocin
5. Progesterone

Q6. Which of the following cells is the predominant (most common) type to be found in the Islets of Langherhans?

1. A - producing glucagon
2. B - producing Insulin
3. D - producing somatostatin
4. F - producing VIP
5. PP - producing substance P

Q7. A 26-year-old lady visits her Doctor for a routine pre-natal visit. If her serum hormones were to be tested, her level of progesterone would be seen to be much higher than in a non-pregnant woman. Where is this progesterone being produced?

1. Adrenal cortex
2. Anterior pituitary
3. Hypothalamus
4. Kidney
5. Ovary


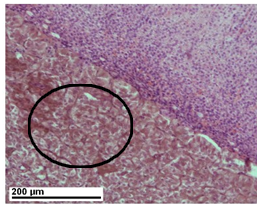
Q8. The polyhedral cells of the adrenal medulla are derived from neuroendocrine cells, and produce amines, like adrenaline and noradrenaline. What histological dye has been used to stain these polyhedral cells (circled) in the image to the right?

1. Eosin
2. Haematoxylin
3. Methylene blue
4. Potassium dichromate
5. Van Gieson’s

Q9. Chromophil cells within the pituitary are so named because they have a good affinity for the colours in certain stains. In sections stained with haematoxylin and eosin, what name is given to the chromophil cells which take up the pink dye?

1. Acidophils
2. Argentaffin cells
3. Basophils
4. Neutrophils
5. Chromaffin cells


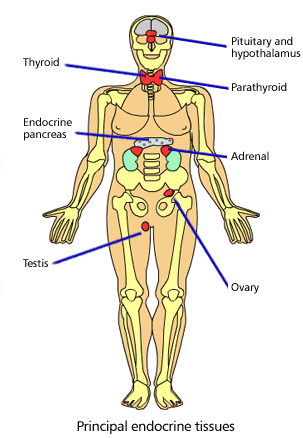
Q10. Multiple endocrine neoplasia is a rare condition that effects endocrine cells derived from the neural crest. Which of the following cells are derived from neuroendocrine cells and so may be involved?

1. Adrenal medulla cells
2. Hepatocytes
3. Juxtaglomerular cells
4. Leydig cells
5. Thyroid follicular cells

Q11. The pituitary gland forms a functional unit with the hypothalamus and has two principal parts, the anterior and posterior pituitary. Which of the following hormones is secreted by the posterior pituitary, or neurohypophysis, which is directly connected to the hypothalamic nuclei?

1. Aldosterone
2. Dopamine
3. Growth hormone
4. Oxytocin
5. Prolactin


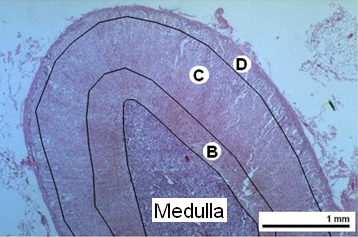
Q12. The adrenal cortex has three distinguishable zones that secrete steroid hormones. Which of the following hormones are secreted by the layer of the cortex labelled “B” in the image to the right?

1. Androgens
2. Catecholamines
3. Glucocorticoids
4. Gonadotrophins
5. Mineralocorticoids

Q13. Within the endocrine system, what is the mode of secretion in which the cell membrane ruptures and the entire contents of the cell are shed?

1. Apocrine
2. Autocrine
3. Holocrine
4. Merocrine
5. Paracrine


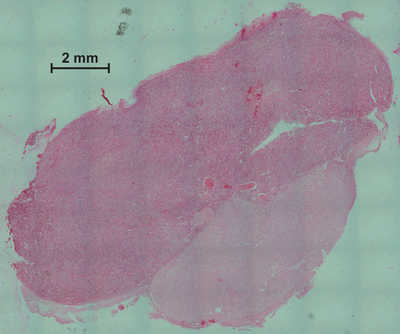
Q14. The image to the right shows shows which of the following glands?

1. Adrenal
2. Pancreas
3. Parathyroid
4. Thyroid
5. Pituitary

**End of Questions**
